# Immunomodulatory role of gut microbial metabolites: mechanistic insights and therapeutic frontiers

**DOI:** 10.3389/fmicb.2025.1675065

**Published:** 2025-09-17

**Authors:** Li Zeng, Yuwei Qian, Xiaoyan Cui, Jingying Zhao, Zhijun Ning, Jinglong Cha, Kun Wang, Changrong Ge, Junjing Jia, Tengfei Dou, Hongyan Chen, Lixian Liu, Zhipeng Bao, Zonghui Jian

**Affiliations:** ^1^Faculty of Animal Husbandry and Veterinary Medicine, Yunnan Vocational and Technical College of Agriculture, Kunming, Yunnan, China; ^2^The Chenggong Department, Kunming Medical University Affiliated Stomatological Hospital, Kunming, Yunnan, China; ^3^Faculty of Animal Science and Technology, Yunnan Agricultural University, Kunming, Yunnan, China; ^4^Institute of Science and Technology, Chuxiong Normal University, Chuxiong City, Yunnan, China

**Keywords:** gut microbiota, metabolites, immune modulation, therapeutic modulation, host-microbiome interactions

## Abstract

The gut microbiota modulates host immunity through a wide array of metabolic products that function as signaling molecules, thereby linking microbial activity with both mucosal and systemic immune responses. Notably, short-chain fatty acids, secondary bile acids, tryptophan-derived indoles, polyamines, and lipid derivatives play pivotal roles in regulating innate and adaptive immune functions via G protein-coupled receptors, nuclear receptors, and epigenetic pathways. These metabolites modulate immune cell differentiation, epithelial barrier integrity, and the resolution of inflammation in a dose- and site-specific manner. Recent advancements in spatial metabolomics, synthetic biology, and nanomedicine have facilitated the spatiotemporal delivery of these immunomodulatory compounds, revealing novel therapeutic avenues for the treatment of inflammatory and autoimmune disorders. This review summarizes the biosynthesis and immunoregulatory functions of key microbial metabolites, highlights the compartmentalized and systemic mechanisms of action, and discusses emerging therapeutic approaches, including postbiotics, engineered probiotics, and receptor-targeting drugs. We also explore the challenges in achieving personalized microbiome-immune modulation and propose future directions integrating multiomics and AI-driven predictive modeling. Understanding the metabolite-immune axis paves the way for novel interventions targeting host-microbe symbiosis.

## Graphical Abstract

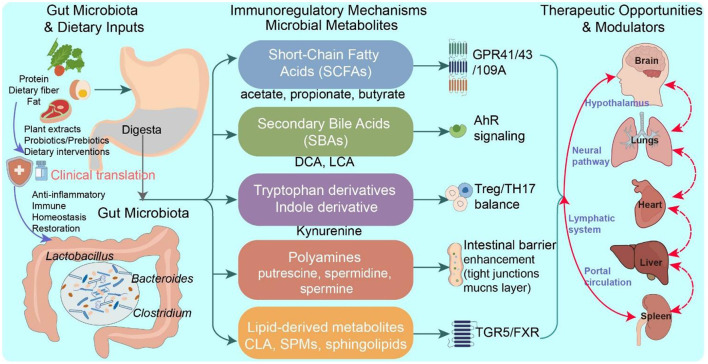

## 1 Introduction

**Figure 1:**
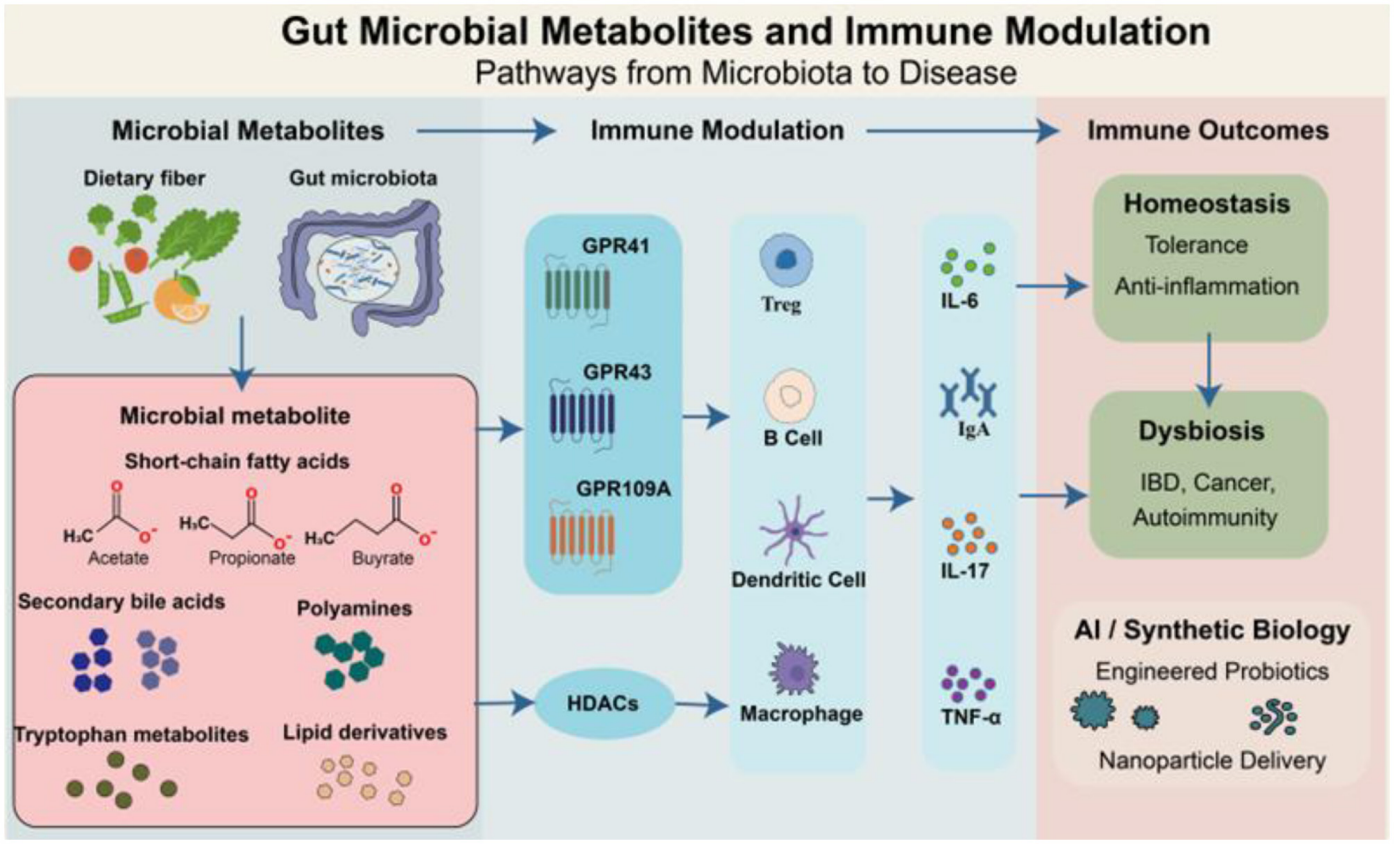
Gut microbial metabolites modulate host immune responses via receptor-mediated and epigenetic pathways. Dietary components are metabolized by the gut microbiota to generate diverse microbial metabolites, including SCFAs, SBAs, polyamines, Trp-derived molecules, and lipid derivatives. These metabolites act as key mediators of host-microbiota interactions, influencing immune responses through G protein-coupled receptors (e.g., GPR41, GPR43, and GPR109A), HDACs, and downstream signaling pathways. These coordinated interactions shape cytokine profiles (e.g., IL-6, IL-17, TNF-α, IgA) and maintain immune homeostasis. In dysbiosis, altered metabolite levels can disrupt immune balance, contributing to diseases such as inflammatory bowel disease (IBD), cancer, and autoimmunity. Advances in synthetic biology and AI-based tools offer novel avenues for microbiota-targeted therapies using engineered probiotics or nanoparticle delivery systems.

Microbial metabolites act as critical mediators in the dynamic interplay between the host and the gut symbiotic microbiota. Owing to their structural diversity and multifunctional properties, these small molecules function as signaling mediators that regulate both mucosal and systemic immune responses. They are predominantly generated through the microbial metabolism of dietary substrates, such as fiber and protein, as well as endogenous compounds such as primary bile acids. These metabolites can be classified into five principal groups: short-chain fatty acids (SCFAs), tryptophan (Trp) derivatives, secondary bile acids (SBAs), polyamines, and lipid-based metabolites (Levy et al., 2017). In addition to serving as essential energy sources for intestinal epithelial cells, these metabolites exert immunomodulatory effects through interactions with host receptors and regulatory pathways, including G protein-coupled receptors (GPR41, GPR43, GPR109A), nuclear receptors (e.g., farnesoid X receptor [FXR], peroxisome proliferator-activated receptor gamma [PPARγ]), and epigenetic modulators such as histone deacetylases (HDACs) (Levy et al., 2016). For example, SCFAs—including acetate, propionate, and butyrate—facilitate regulatory T (Treg) cell differentiation and reinforce intestinal barrier integrity via the activation of GPR41/43 and the inhibition of HDACs (Kim, 2021; Liu et al., 2023). Indole-3-propionic acid (IPA), a microbial Trp-derived metabolite, enhances mucosal defense mechanisms through the activation of the aryl hydrocarbon receptor (AhR)-IL-22 signaling pathway (Hezaveh et al., 2022). SBAs influence the immune phenotype of hepatic Kupffer cells and modulate inflammasome activation through FXR and Takeda G-protein-coupled bile acid receptor 5 (TGR5) signaling (Hao et al., 2017). Polyamines influence T-cell lineage commitment by regulating autophagy and metabolic reprogramming (Stewart et al., 2022). Additionally, conjugated linoleic acid (CLA) exerts anti-inflammatory effects by suppressing proinflammatory cytokine expression through the PPARγ/NF-κB signaling cascade (Kennedy et al., 2009) (Figure 1).

Disruptions in the gut microbial balance, or dysbiosis, lead to significant alterations in both the production and spatial distribution of microbial metabolites, often culminating in immune imbalance (Yang et al., 2018; Mahalingam and Pandiyan, 2024). For example, a decrease in butyrate-producing microbes weakens the inhibition of HDAC, thereby contributing to the pathogenesis of inflammatory bowel disease. In parallel, insufficient availability of AhR ligands impairs the equilibrium between Tregs and Th17 cells, increasing the risk of autoimmune disorders (Hezaveh et al., 2022). Moreover, increased levels of certain secondary bile acids, such as deoxycholic acid (DCA), are associated with genotoxic stress and the activation of pro-oncogenic signaling cascades (Holtmann et al., 2021).

Rapid advancements in spatial metabolomics, single-cell omics, and synthetic biology have enabled researchers to map the *in situ* localization of gut metabolites with high resolution and elucidate their roles within immune regulatory circuits. Concurrently, advances in deep learning and artificial intelligence are enhancing the ability to predict metabolite-receptor interaction networks, thereby supporting the rational design of engineered probiotics and precision-targeted delivery systems (Mar et al., 2023; Wang M. et al., 2023). The convergence of metagenomic, metabolomic, and immune phenotyping datasets with AI-based predictive modeling offers significant potential for unraveling the complex regulatory landscape of the “metabolite-immune axis.” This integrative framework may drive the development of innovative therapeutic interventions and translational applications across inflammatory, autoimmune, and oncological disorders (Figure 1).

## 2 The biosynthesis of intestinal microbial metabolites and the mechanism of immune regulation

Gut microbial metabolites, as pivotal mediators of host-microbiota interactions, encompass five major classes with notable immunomodulatory potential: SCFAs, SBAs, tryptophan-derived metabolites, polyamines, and lipid mediators. These compounds are generated through the microbiota's selective transformation of dietary components—such as dietary fibers, proteins, and bile acids—via biosynthetic pathways finely regulated by microbial composition, intestinal pH, and the host's nutritional status. Critically, they are deeply involved in essential physiological processes, including the reinforcement of mucosal barriers, the differentiation of immune cell subsets, and the resolution of inflammation. To provide a systematic overview of the biosynthetic networks of these metabolites and their central roles in immune regulation, a detailed summary is presented in Table 1.

**Table 1:** Summary of key gut microbial metabolites: biosynthesis and immunoregulatory mechanisms.

**Metabolites**	**Key points of biosynthesis**	**Key points of immunoregulatory mechanisms**
Short-chain fatty acids (SCFAs) (acetate, propionate, butyrate)	Produced by fermentation of dietary fiber by anaerobic bacteria such as *Clostridium* and *Bacteroides* (Fernandes et al., 2014; Litvak et al., 2018; Krautkramer et al., 2021; den Besten et al., 2013) Pathways include glycolysis, pentose phosphate pathway, butyrate kinase pathway (Kim, 2021; Krautkramer et al., 2021) Ratio typically ~3:1:1 in healthy colon (Fernandes et al., 2014)	Activate GPR41/43/109A and inhibit HDACs to modulate immune cell differentiation (Kim, 2021; Yang et al., 2020; Noureldein and Eid, 2018) Promote Treg induction and anti-inflammatory cytokine production (IL-10, TGF-β) (Furusawa et al., 2013a) Enhance epithelial barrier integrity, mucus secretion, and IgA production (Lin et al., 2015; Kim et al., 2016)
Secondary bile acids (SBAs) (DCA, LCA)	Derived from hepatic primary bile acids via bacterial 7α-dehydroxylation (*Clostridium, Bacteroides*) (Vico-Oton et al., 2024; Ridlon et al., 2023) Enzymes encoded in bai operon (e.g., *BaiCD, BaiE*) (Ridlon et al., 2023) Profile influenced by diet, pH, microbiota composition, and host FXR-FGF15/19 feedback (Zhang et al., 2022; Chinda et al., 2022)	Activate FXR, TGR5, and AhR to regulate cytokine expression and barrier function (Kim et al., 2024; Zhou et al., 2022; Anderson and Gayer, 2021) Modulate Treg/Th17 balance (e.g., 3-oxoLCA inhibits Th17; isoalloLCA promotes Tregs) (Cai et al., 2022; Fu et al., 2025) Enhance IgA-mediated mucosal immunity (Beller et al., 2020)
Tryptophan-derived metabolites (indole-3-propionic acid, indole-3-aldehyde, kynurenine)	Three main pathways: indole, kynurenine, serotonin (enterochromaffin cells) (Stone and Williams, 2024) Produced by *Lactobacillus, Bifidobacterium*, and host enzymes IDO1/TDO (Wang Y. et al., 2023; Wang et al., 2025) Influenced by dietary Trp and microbiota composition (Stone and Williams, 2024; Ye et al., 2022)	Indole derivatives activate AhR to promote IL-22 secretion, Treg induction, and M2 macrophage polarization (Wang M. et al., 2023; Wang et al., 2025; Ye et al., 2022) Kynurenine pathway metabolites modulate macrophages via GPR35 (Sun et al., 2022) Maintain mucosal defense and limit excessive inflammation (Ye et al., 2022; Alli et al., 2022)
Polyamines (putrescine, spermidine, spermine)	Synthesized from arginine/ornithine via arginine decarboxylase and ornithine decarboxylase (Tofalo et al., 2019; Ramos-Molina et al., 2019) Produced by host cells and gut microbes such as *Bacteroides fragilis, Enterococcus faecalis* (Ramos-Molina et al., 2019) Influenced by diet, pH, and inflammation (Tofalo et al., 2019)	Regulate macrophage and dendritic cell polarization (Zhang et al., 2019; Chamoto et al., 2024) Control T-cell lineage fate via metabolic reprogramming and eIF5A hypusination (Zhang et al., 2019) Enhance memory B-cell function and antibody production (Zhang et al., 2019)
Lipid-derived metabolites (conjugated linoleic acids)	CLA from diet or microbial biotransformation (*Lactobacillus, Bifidobacterium*) (Chen R. et al., 2025) SPMs from ω-3 fatty acids via lipoxygenase, cyclooxygenase, P450 pathways (Beyer et al., 2023) Sphingolipids from host and microbes (*Bacteroides fragilis*) (Bai et al., 2023)	CLAs modulate NF-κB and PPARγ signaling (Chen Y. et al., 2025) SPMs resolve inflammation by inhibiting neutrophil infiltration and promoting efferocytosis (Maliha et al., 2024; de Souza et al., 2025) Sphingolipids regulate Th17/Treg balance and TLR4-mediated inflammation (Zou et al., 2023; Lee et al., 2023)

### 2.1 SCFAs

#### 2.1.1 Biosynthesis of SCFAs

SCFAs are key end products of dietary fiber fermentation by the gut microbiota and play a central role in host-microbe interactions. Their synthesis is strongly influenced by microbial diversity, host nutritional habits, and the intestinal milieu. In healthy individuals, the main SCFAs—acetate, propionate, and butyrate—are produced at a typical ratio of about 3:1:1 (Fernandes et al., 2014). This proportion, however, can shift markedly in response to high-fat diets, advancing age, or pathological conditions. The colonic environment maintains a state of low oxygen, shaped by epithelial oxygen consumption and the activity of facultative anaerobes, creating conditions that support the dominance of obligate anaerobes such as species of *Clostridium* and *Bacteroides* (Litvak et al., 2018). These bacteria possess an extensive suite of carbohydrate-active enzymes, including glycoside hydrolases, along with key metabolic enzymes such as succinate decarboxylase and acetyl-CoA transferase. This enzymatic capacity enables them to degrade complex dietary polysaccharides into simple sugars and convert them into SCFAs via pathways such as glycolysis, the pentose phosphate pathway, and related anaerobic metabolic routes (Kim, 2021; Krautkramer et al., 2021). The intestinal pH plays a critical regulatory role in SCFA synthesis by shaping the microbial composition and modulating enzyme activity. For example, at pH 5.5, butyrate-producing bacteria such as *Faecalibacterium prausnitzii* become dominant, whereas at pH 6.5, genera such as *Bacteroides* and *Bifidobacterium* preferentially produce acetate and propionate (den Besten et al., 2013).

SCFA biosynthetic pathways exhibit notable species specificity. *Akkermansia muciniphila* and *Prevotella* spp. are key acetate producers that activate GPR43-mediated signaling. *Phascolarctobacterium succinatutens* and *Veillonella* spp. generate propionate predominantly via the succinate pathway, whereas butyrate is synthesized mainly by *Coprococcus eutactus* and related taxa through the butyrate kinase pathway (Liu et al., 2023). SCFAs exhibit a steep concentration gradient throughout the colon, with levels reaching 70–140 mM in the proximal colon and decreasing to 20–70 mM in the distal segments (Cummings et al., 1987). Dietary intake directly influences SCFA production. High-fiber diets promote the proliferation and metabolic flux of SCFA-producing microbes (den Besten et al., 2013).

#### 2.1.2 Immunomodulatory mechanisms of SCFAs

**Figure 2:**
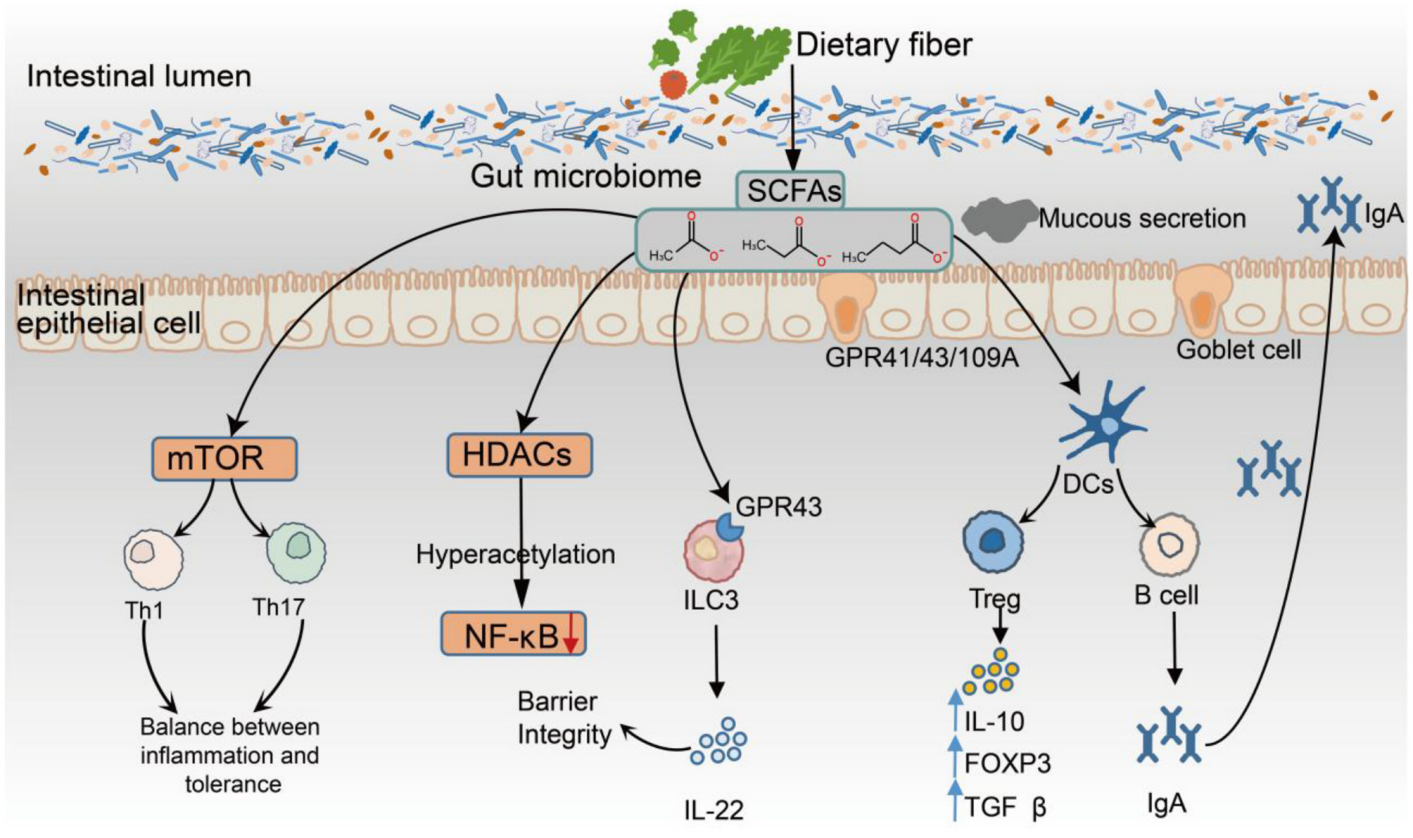
Immunomodulatory Mechanisms of SCFAs in the Gut Environment. SCFAs, produced by microbial fermentation of dietary fiber, exert multifaceted effects on the intestinal immune system through three primary mechanisms: (1) activation of GPR41, GPR43, and GPR109A; (2) inhibition of HDACs, leading to epigenetic remodeling; and (3) regulation of cellular metabolism via the mTOR signaling pathway. SCFAs enhance epithelial barrier integrity by modulating NF-κB activity and promoting IL-22 secretion from ILC3s expressing GPR43. Butyrate and propionate suppress the production of proinflammatory cytokines (e.g., IL-6 and TNF-α) via HDAC inhibition and facilitate the differentiation of regulatory Tregs by inducing FOXP3 expression and upregulating the anti-inflammatory cytokines IL-10 and TGF-β. In addition, SCFAs promote B-cell differentiation and enhance mucosal IgA production. They also modulate the phenotype of antigen-presenting cells, supporting immune tolerance and limiting excessive activation of Th1 and Th17 responses.

SCFAs exert broad immunomodulatory effects that are vital for preserving mucosal immune equilibrium. These metabolites act through diverse mechanisms, including the activation of G_protein-coupled receptors (GPR41, GPR43, and GPR109A), the inhibition of HDAC as epigenetic regulators, and the modulation of key metabolic pathways, such as mTOR signaling, thereby influencing immune cell differentiation, activation, and function (Yang et al., 2020; Noureldein and Eid, 2018) (Figure 2).

Within the innate immune system, SCFAs support epithelial defenses by promoting the production of IL-22 from group 3 innate lymphoid cells (ILC3s) via GPR43 signaling, which enhances mucus secretion and increases barrier integrity (Lin et al., 2015). Moreover, SCFAs inhibit NF-κB signaling through HDAC suppression, leading to reduced proinflammatory cytokine production and the maintenance of homeostatic macrophage and DC phenotypes (Ganapathy et al., 2013). In adaptive immunity, SCFAs significantly influence T-cell differentiation pathways. Butyrate, in particular, promotes the development of regulatory Tregs by upregulating FOXP3 expression and inducing anti-inflammatory cytokines such as IL-10 and TGF-β through HDAC inhibition (Furusawa et al., 2013b), thereby reinforcing immune tolerance. Moreover, SCFAs can support moderate activation of Th1 and Th17 effector subsets through mTOR-dependent signaling, promoting a balanced immune response (Kim, 2021). In the central nervous system, butyrate facilitates the polarization of microglia toward a homeostatic phenotype (P2RY12^+^, TMEM119^+^) via HDAC inhibition, whereas propionate modulates microglial cytokine output through GPR43, downregulating the expression of IL-6 and IL-1β. These effects have been associated with attenuated neuroinflammation and decreased amyloid-beta plaque burden in Alzheimer's disease models (Lan et al., 2024; Caetano-Silva et al., 2023). SCFAs also modulate the phenotype of dendritic cells (DCs) and macrophages, leading to reduced expression of costimulatory molecules and IL-12, which limits the activation of proinflammatory T cells (Frikeche et al., 2012). In humoral immunity, SCFAs promote the differentiation of B cells into IgA-producing plasma cells, thereby increasing antibody secretion and reinforcing mucosal immune defense (Kim et al., 2016). Collectively, SCFAs coordinate innate and adaptive immune responses by integrating receptor-mediated signaling, epigenetic modulation, and metabolic reprogramming. Their multifaceted immunoregulatory functions are now recognized as key components of the host-microbiota interface, underscoring their central role in maintaining immune homeostasis (Figure 2).

### 2.2 SBAs

#### 2.2.1 Biosynthesis of SBAs

SBAs are predominantly generated in the gut through microbial conversion of liver-synthesized primary bile acids (PBAs), primarily via 7α-dehydroxylation catalyzed by anaerobic bacteria, notably *Clostridium* and *Bacteroides* spp. (Vico-Oton et al., 2024). Key SBAs include DCA and lithocholic acid (LCA) (Ridlon et al., 2023). This biotransformation is facilitated by enzymes encoded within the bai operon, particularly *BaiCD* and *BaiE*, and is modulated by factors such as substrate availability, luminal pH, oxygen gradients, and the presence of cometabolites such as SCFAs. Following their synthesis, SBAs can be reabsorbed into the enterohepatic circulation via intestinal transporters, including the apical sodium-dependent bile acid transporter and the organic solute transporter α/β, thereby contributing to the regulation and stability of the systemic bile acid pool (Zhang et al., 2022). The composition and spatial distribution of SBAs vary greatly between individuals, with the highest concentrations typically observed in the ileum and ascending colon, highlighting their site-specific metabolism. Dietary habits, antibiotic use, and probiotic interventions significantly shape SBA profiles by modulating the abundance of 7α-dehydroxylating bacteria. For example, high-fat diets promote DCA accumulation, whereas antibiotic exposure suppresses these microbes, leading to reduced SBA levels (Chinda et al., 2022). Moreover, hepatic enzymes such as *CYP7A1* and the ileal fibroblast growth factor 15/19–FXR axis exert negative feedback control over SBA synthesis, underscoring their central role in gut-liver signaling.

#### 2.2.2 Immunomodulatory mechanisms of SBAs

**Figure 3:**
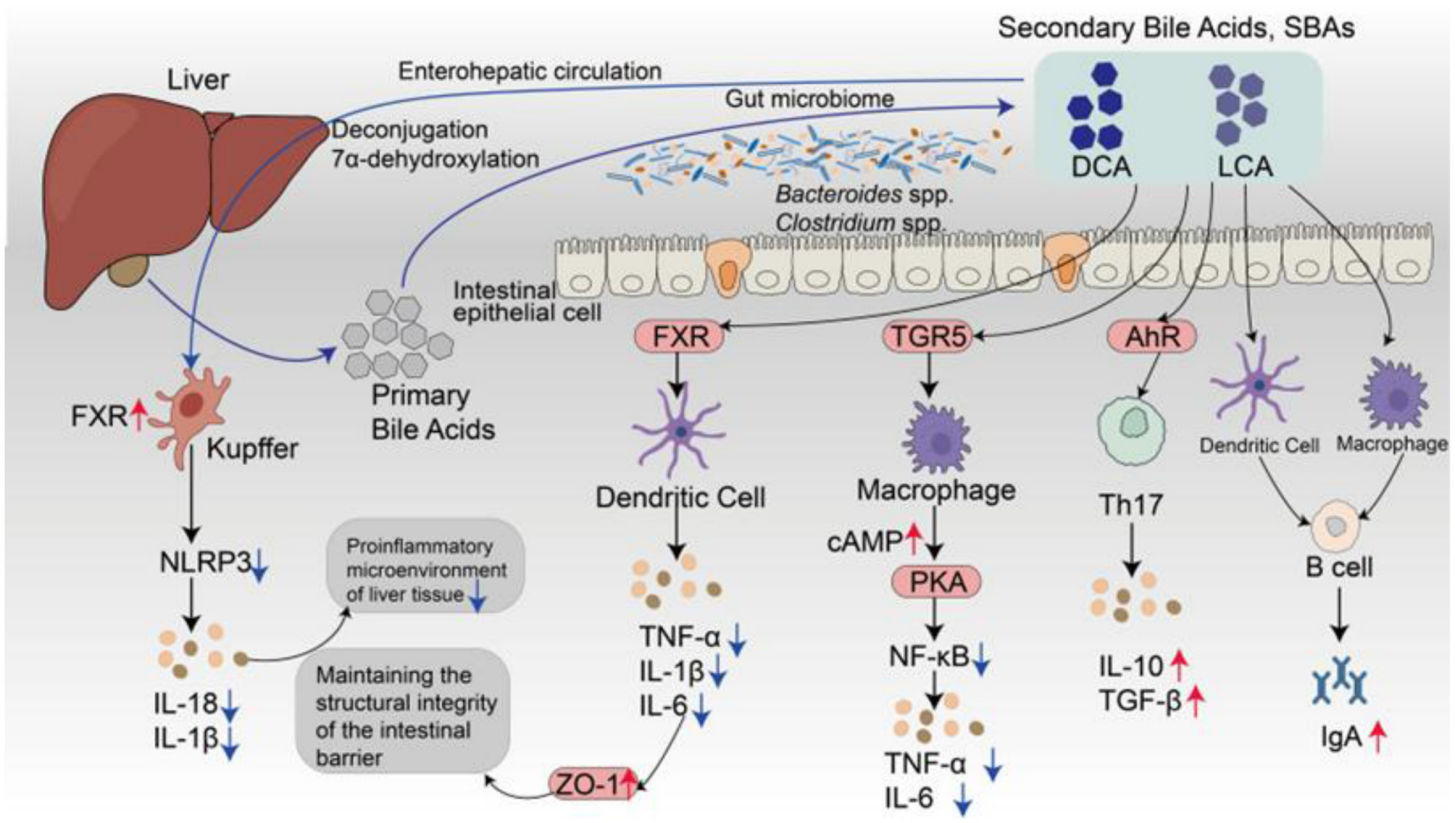
Schematic illustration of gut-liver-immune regulation mediated by SBAs. PBAs are synthesized in the liver and secreted into the intestinal lumen, where they are transformed by the gut microbiota, primarily *Clostridium* and *Bacteroides* species, into SBAs, such as DCA and LCA, via microbial 7α-dehydroxylation. These SBAs are reabsorbed into enterocytes through active transporters, including the acid transporter and the organic solute transporter organic solute transporter α/β, and re-enter the liver via the portal vein as part of the enterohepatic circulation. As bioactive signaling molecules, SBAs function as ligands for both nuclear and membrane receptors, such as FXR, TGR5, and AhR, which are differentially expressed across epithelial cells, hepatic Kupffer cells, DCs, and macrophages. In both intestinal and hepatic immune cells, FXR activation suppresses NLRP3 inflammasome assembly and dampens the production of proinflammatory cytokines, including IL-1β, IL-6, and TNF-α. FXR also strengthens epithelial barrier integrity by promoting the expression of tight junction proteins such as ZO-1. In macrophages, TGR5 activation enhances cyclic adenosine monophosphate/protein kinase A signaling, thereby attenuating NF-κB-driven inflammatory responses. AhR ligands derived from SBAs further regulate adaptive immunity by promoting regulatory Treg differentiation and enhancing the secretion of IL-10 and TGF-β while concurrently suppressing Th17 polarization. These convergent signaling pathways collectively contribute to mucosal immune tolerance and support the activation of IgA^+^ B cells, thereby promoting intestinal immune homeostasis.

In addition to their metabolic functions, SBAs serve as bioactive signaling molecules that modulate immune responses along the gut-liver-immune axis. Their immunoregulatory actions are primarily mediated through the activation of nuclear and membrane-bound receptors, including FXR, TGR5, and AhR (Figure 3). These receptors are expressed across multiple cell types—such as intestinal epithelial cells, hepatic Kupffer cells, macrophages, and DCs—enabling SBAs to influence both innate and adaptive immune processes (Kim et al., 2024; Zhou et al., 2022). FXR activation suppresses the expression of proinflammatory cytokines such as TNF-α, IL-6, and IL-1β, enhances the expression of tight junction proteins (e.g., zonula occludens-1[ZO-1]), and increases epithelial barrier integrity. In liver-resident Kupffer cells, FXR also inhibits NLRP3 inflammasome activation, thereby alleviating hepatic inflammation (Anderson and Gayer, 2021). TGR5 stimulation elevates intracellular cyclic adenosine monophosphate levels via the protein kinase A pathway, which in turn suppresses NF-κB signaling and proinflammatory cytokine release by macrophages (Shi et al., 2021). TGR5 also promotes the secretion of metabolic hormones such as glucagon-like peptide-1, suggesting a link between immune regulation and metabolic control. LCA and its derivatives enhance mucosal immunity by increasing ILC3 function and promoting IL-22-mediated epithelial defense (Bao et al., 2024; Qian et al., 2025).

Within the adaptive immune system, SBAs modulate CD4^+^ T-cell differentiation, particularly by influencing the equilibrium between Tregs and Th17 cells. For example, 3-oxo LCA suppresses Th17 cell differentiation by inhibiting the transcription factor retinoid-related orphan receptor gamma, whereas heteroallelic isoalloLCA promotes Treg induction by enhancing TGF-β signaling through reactive oxygen species-dependent mechanisms (Cai et al., 2022; Fu et al., 2025). Additionally, SBAs function as endogenous ligands for AhR, facilitating immune tolerance by upregulating the expression of IL-10 and TGF-β (Sharma et al., 2025). These immunoregulatory pathways may operate in concert with SCFA-mediated HDAC inhibition to maintain the balance between proinflammatory and immunosuppressive responses (Du et al., 2024). Importantly, SBAs also contribute to humoral immune regulation by promoting crosstalk between Tregs and DCs within Peyer's patches, thereby increasing the activation of IgA-producing B cells and supporting mucosal antibody production (Beller et al., 2020). Overall, SBAs are increasingly recognized not as mere metabolic byproducts but as pivotal immunometabolic modulators that coordinate host-microbiota signaling and immune homeostasis.

### 2.3 Trp-derived metabolites

#### 2.3.1 Biosynthesis of Trp

Trp metabolism plays a critical role in regulating host immune responses and preserving microbial equilibrium. Trp-derived metabolites are produced through three principal metabolic pathways: (1) the indole pathway, which is primarily mediated by the gut microbiota; (2) the kynurenine pathway, which predominantly occurs in host cells; and (3) the serotonin or 5-hydroxytryptamine pathway, which is mainly active in gut enterochromaffin cells. These pathways yield a variety of bioactive compounds—such as indole-3-aldehyde (IAld), kynurenic acid, and 5-hydroxytryptamine—that engage immunologically relevant receptors, including AhR, GPR35, and the general control nonderepressible 2 kinase. Through these interactions, Trp metabolites contribute to both mucosal and systemic immune modulation (Stone and Williams, 2024). The kynurenine pathway is initiated by the enzymatic action of indoleamine 2,3-dioxygenase or Trp 2,3-dioxygenase, which converts Trp into kynurenine, leading to the formation of downstream immunoregulatory metabolites such as kynurenic acid and 3-hydroxykynurenine (Yang et al., 2024).

#### 2.3.2 Immunomodulatory mechanisms of Trp

**Figure 4:**
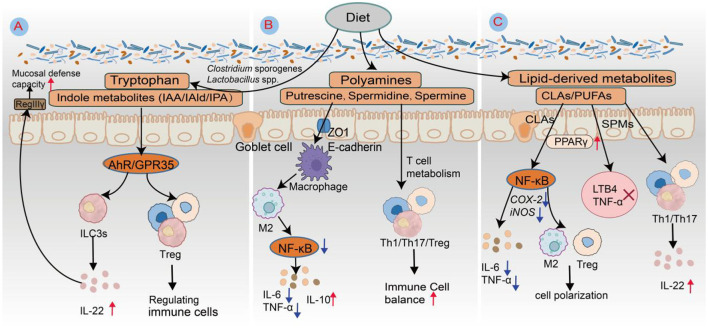
Schematic diagram of the roles of Trp metabolites, polyamines, and lipid-derived mediators in intestinal immune regulation. **(A)** Trp-derived metabolites. Anaerobic gut bacteria such as *Clostridium sporogenes, Lactobacillus* spp., and *Bacteroides* spp. metabolize Trp via decarboxylation or transamination pathways to produce indole derivatives, including IAA, IAld, and IPA. These metabolites serve as endogenous ligands for AhR, activating ILC3s to secrete IL-22. IL-22, in turn, stimulates intestinal epithelial cells to express antimicrobial peptides such as RegIIIγ, thereby enhancing mucosal defense against pathogenic invasion. **(B)** Polyamines. Spermidine, a representative polyamine metabolite, promotes the polarization of macrophages toward the M2 anti-inflammatory phenotype. This effect is mediated through the inhibition of the NF-κB signaling pathway, resulting in the downregulation of the proinflammatory cytokines IL-6 and TNF-α and the upregulation of the anti-inflammatory cytokine IL-10. Consequently, spermidine contributes to the establishment of an immunosuppressive microenvironment conducive to tissue repair. **(C)** Lipid-derived mediators. CLA activates PPARγ signaling while suppressing NF-κB pathway activity. This dual modulation reduces the expression of inflammatory enzymes such as cyclooxygenase-2 and inducible nitric oxide synthase (iNOS), inhibits the release of proinflammatory cytokines, including IL-6 and TNF-α, and promotes regulatory T-cell (Treg) expansion as well as macrophage M2 polarization. Metabolites derived from eicosapentaenoic acid, resolvin E1 (RvE1), and resolvin E2 (RvE2) engage the CMKLR1 receptor to suppress leukotriene B4 (LTB4) and TNF-α production. Moreover, docosahexaenoic acid-derived resolvins (RvD1–RvD3) regulate T-cell fate via GPR32 and GPR18, inhibiting Th1 and Th17 responses while enhancing Treg functionality to maintain immune homeostasis.

Trp metabolites modulate immune responses predominantly through receptor-mediated signaling, with particular emphasis on AhR and GPR35. Among these metabolites, indole derivatives function as prominent endogenous ligands for AhR, exerting immunoregulatory effects by influencing NF-κB signaling, NLRP3 inflammasome activation, immune cell polarization, and T-cell differentiation (Figure 4A). For example, IAld suppresses NF-κB activation and inhibits NLRP3 inflammasome assembly, reducing proinflammatory cytokine production (e.g., IL-6 and TNF-α) and ameliorating colonic inflammation in DSS-induced colitis models (Wang M. et al., 2023). Indole-3-acetic acid, a metabolite produced by *Lactobacillus* species, contributes to immune modulation by driving macrophage polarization toward the anti-inflammatory M2 phenotype and stimulating the secretion of interleukin-10 (IL-10) (Wang et al., 2025). Metabolites from the kynurenine pathway also possess significant immunoregulatory functions. For example, kynurenic acid interacts with GPR35 to control macrophage responses by reducing calcium influx and limiting the production of reactive oxygen species, thereby suppressing NLRP3 inflammasome activation and the maturation of IL-1β under inflammatory conditions (Sun et al., 2022). Additionally, indole-derived compounds such as IAld and IPA support the proliferation of FoxP3^+^ regulatory Tregs and inhibit Th17 cell differentiation through AhR signaling (Ye et al., 2022). These immunological effects are further amplified in the presence of dietary AhR ligands, including quercetin and resveratrol, highlighting a coordinated microbiota-diet-AhR regulatory axis. In addition to AhR-dependent pathways, kynurenine metabolites also exert immune-modulating effects through other signaling mechanisms. For example, kynurenic acid, by engaging GPR35, inhibits inflammasome assembly in macrophages, thereby attenuating both intestinal and systemic inflammation (Sun et al., 2022). In the tumor microenvironment, the overexpression of indoleamine 2,3-dioxygenase 1 (IDO1) reprograms Trp metabolism toward kynurenine synthesis, depleting local Trp pools. This activates the general control non-derepressible 2 kinase, which induces metabolic stress and functional exhaustion in T cells, facilitating immune evasion (Xing et al., 2024). Concurrently, IDO1 activation enhances Treg induction via the AhR-TGF-β1 axis, which cooperates with M2 tumor-associated macrophages to construct a tolerogenic immune niche (Seo and Kwon, 2023). In autoimmune diseases such as systemic lupus erythematosus, patients often exhibit elevated plasma Kyn/Trp ratios, accompanied by increased Th17 cell frequencies, decreased Treg populations, and the upregulation of proinflammatory cytokines such as IL-17 and IL-6—indicative of sustained kynurenine pathway hyperactivation and immune tolerance breakdown (Joanna et al., 2024; Huang et al., 2024). Notably, probiotic supplementation (e.g., *Bifidobacterium* spp.) can partially normalize the Kyn/Trp ratio, suggesting that microbiota-kynurenine pathway axis modulation may offer promising avenues for nutritional immunotherapy (Alli et al., 2022).

### 2.4 Polyamines

#### 2.4.1 Biosynthesis of polyamines

Polyamines are small, positively charged metabolites produced by both host cells and the gut microbiota. The primary polyamines—putrescine, spermidine, and spermine—are synthesized predominantly through the arginine-ornithine pathway. In this pathway, arginine is initially converted to putrescine via the action of arginine decarboxylase, followed by the stepwise generation of spermidine and spermine through an S-adenosylmethionine-dependent mechanism. Ornithine decarboxylase serves as the rate-limiting enzyme in this biosynthetic route and is regulated by intracellular signaling pathways such as *Myc* and *mTOR* (Tofalo et al., 2019). The production of polyamines by gut microbes is influenced by both the microbial composition and the availability of precursor metabolites. Certain commensal bacteria—including *Bacteroides fragilis, Enterococcus faecalis*, and *Clostridium* species—possess distinct arginine decarboxylase and ornithine decarboxylase systems that significantly contribute to the gut's polyamine reservoir (Ramos-Molina et al., 2019). Environmental variables, including the intestinal pH, inflammatory conditions, and dietary factors—particularly protein-rich or polyamine-rich diets—also play critical roles in modulating polyamine synthesis and turnover.

#### 2.4.2 Immunomodulatory mechanisms of polyamines

Polyamines are widely distributed in the gastrointestinal tract, lymphoid organs, and central nervous system, where they play essential roles in immune regulation, inflammation control, and antitumor immune responses (Nair et al., 2025). In the innate immune system, these molecules influence the functional polarization of macrophages and DCs, thereby modulating cytokine expression and inflammatory signaling (Figure 4B). In experimental autoimmune encephalomyelitis, a murine model of neuroinflammation, the administration of spermidine has been shown to reduce the infiltration of CD4^+^ T cells into the central nervous system and alleviate associated symptoms. This effect is partly mediated by enhancing the differentiation of M2-type macrophages with anti-inflammatory properties and limiting the recruitment of proinflammatory myeloid populations (Zhang et al., 2019; Chamoto et al., 2024). By increasing the expression of tight junction proteins such as ZO-1 and claudins, spermidine also strengthens gut barrier integrity, which helps prevent microbial components from activating toll-like receptor (TLR)-driven innate immune responses (Nakamura and Matsumoto, 2025).

In adaptive immunity, polyamines are crucial for metabolic reprogramming and the differentiation of T-cell lineages. Targeted deletion of the *Odc* gene in T cells leads to imbalances among Th1, Th17, and Treg subsets, underscoring their role in determining T-cell fate (Puleston et al., 2021). Notably, spermidine facilitates the hypusination of eukaryotic initiation factor 5A, a post-translational modification necessary for efficient protein synthesis in activated T cells, thereby supporting memory T-cell development and effector functions. Inhibition of this pathway promotes the conversion of Th17 cells into Tregs, highlighting a potential avenue for treating autoimmune conditions such as inflammatory bowel disease and rheumatoid arthritis. In B cells, spermidine enhances immune activity via a distinct route by promoting eukaryotic initiation factor 5A-mediated translation of the transcription factor EB, which drives autophagy, antigen presentation, and antibody production. Importantly, spermidine supplementation has been found to restore memory B-cell function and increase IgG levels in aged mice, suggesting its potential for enhancing vaccine efficacy and rejuvenating immune responses (Zhang et al., 2019).

### 2.5 Lipid-derived metabolites

#### 2.5.1 Biosynthesis of lipid-derived

Lipid-derived metabolites constitute crucial mediators in the crosstalk between the gut microbiota and the host immune system. Current research focuses primarily on CLAs, specialized pro-resolving mediators (SPMs) derived from ω-3 polyunsaturated fatty acids, and sphingolipid molecules, revealing their unique roles in regulating innate and adaptive immunity as well as their associations with disease (Figure 4C). CLAs represent a group of naturally occurring linoleic acid isomers predominantly found in the milk fat of ruminants, with cis-9, trans-11 and trans-10, cis-12 being the principal isomers. Certain strains of *Lactobacillus* and *Bifidobacterium* possess CLA isomerase enzymes capable of converting linoleic acid into immunomodulatory CLA isomers (Chen R. et al., 2025). These CLAs may be acquired either through dietary sources or via microbial biotransformation within the gastrointestinal tract. SPMs—including resolvins (RvD1–RvD4, RvE1–RvE3), protectins, and maresins—are synthesized intracellularly from omega-3 fatty acids such as eicosapentaenoic acid and docosahexaenoic acid, which is mediated by the coordinated activity of lipoxygenases, cyclooxygenases, and cytochrome P450 enzymes (Beyer et al., 2023). Sphingolipid metabolites, including ceramides and sphingosine-1-phosphate (S1P), are endogenously produced by the host but can also be synthesized by gut commensals such as *B. fragilis* and *Lactobacillus spp*., which generate sphingolipids containing α-sphingosine backbones. These microbe-derived sphingolipids modulate host sphingolipid metabolic pathways and contribute to the regulation of immune microenvironment homeostasis (Bai et al., 2023).

#### 2.5.2 Immunomodulatory mechanisms of lipid-derived compounds

Lipid metabolites serve as crucial mediators in the dynamic interaction between the gut microbiota and the host immune system, playing a central role in mucosal immune regulation, immune cell polarization, and the resolution and repair of inflammation (Figure 4C). CLAs predominantly exist as cis-9, trans-11 and trans-10, cis-12 isomers; the former is associated with anti-inflammatory effects, whereas the latter may elicit mild proinflammatory responses under specific conditions (Chen R. et al., 2025). Polyunsaturated fatty acids, such as eicosapentaenoic acid and docosahexaenoic acid, undergo enzymatic conversion into SPMs—a class of bioactive lipid compounds that includes resolvins, protectins, and maresins (Maliha et al., 2024). These SPMs not only suppress inflammatory processes but also actively facilitate their resolution and support tissue regeneration. Mechanistically, SPMs inhibit neutrophil chemotaxis, promote macrophage-mediated efferocytosis of apoptotic cells, and increase IL-22 production, thereby contributing to the restoration of intestinal barrier integrity (Figure 4C). Preclinical studies have demonstrated that SPM-enriched fish oil formulations improve recovery from exercise-induced tissue injury and accelerate organ function restoration in critically ill patients. Nevertheless, challenges remain for clinical translation owing to high dosage requirements, metabolic instability, and difficulties in targeted delivery (de Souza et al., 2025).

Sphingolipid metabolites, including ceramides S1P and their derivatives, have garnered increasing attention as microbial lipid products. Certain microbe-derived ceramide isoforms attenuate lipopolysaccharide (LPS)-induced proinflammatory responses by inhibiting the TLR4-MyD88 signaling pathway. S1P regulates the migratory balance of Th17 cells and Tregs via its receptor S1PR1, exerting a significant influence on inflammatory bowel disease and tumor microenvironment-mediated immune tolerance (Zou et al., 2023). Evidence suggests that S1P signaling promotes tumor-associated M2 macrophage polarization and Treg recruitment, thereby exacerbating immunosuppressive conditions (Lee et al., 2023). Although their immunomodulatory potential is increasingly recognized, their structural complexity, diverse molecular targets, and incomplete understanding of the *in vivo* metabolism of sphingolipid metabolites currently limit their practical application in precision immunotherapy.

## 3 Specific mechanisms of metabolism-immune interactions

### 3.1 Local regulation at the intestinal mucosal interface

The intestinal mucosal interface represents a key site of interaction between microbial metabolites and the host immune system and is characterized by pronounced spatial, temporal, and cell type-specific regulatory dynamics. Intestinal epithelial cells facilitate the transmembrane transport of SCFAs and Trp-derived metabolites via specialized transporters, including monocarboxylate transporters (MCT1/MCT4) and organic cation/carnitine transporters. These metabolites modulate the phosphorylation of tight junction proteins such as claudins, thereby reinforcing mucosal barrier integrity and restricting the translocation of pathogens and toxins (Zhang et al., 2023). SCFAs exhibit concentration gradients along the gastrointestinal tract, leading to region-specific functional outcomes. In the proximal colon, elevated butyrate concentrations inhibit HDAC activity, thereby increasing tight junction protein expression and preserving epithelial integrity. In contrast, in the distal colon, reduced butyrate levels activate GPR43, stimulating goblet cell mucus secretion, which bolsters the physical barrier and enhances epithelial defense and repair (Liu et al., 2023). Innate immune cells within the lamina propria display heightened sensitivity to these localized metabolic signals. Under the influence of SCFAs, plasma cells assume critical immunological functions; propionate and other SCFAs upregulate X-box binding protein 1 and B lymphocyte-induced maturation protein 1 (Blimp-1) via HDAC inhibition, thereby promoting IgA secretion and J-chain-mediated polymerization. This enhances IgA transcytosis and supports the immune exclusion of luminal pathogens (Saleri et al., 2022). Moreover, butyrate restricts excessive proliferation within Lgr5^+^ intestinal stem cell niches by modulating the Wnt/β-catenin signaling axis, thereby maintaining epithelial homeostasis. Conversely, Trp-derived metabolites activate mechanistic target of rapamycin complex 1 signaling at the villus tip, facilitating epithelial regeneration and promoting dynamic mucosal repair and barrier preservation (Zhang et al., 2023). Leveraging this spatially nuanced regulatory landscape, recent advances in synthetic biology have led to the development of “smart engineered probiotics.” These innovations incorporate CRISPR-dCas9 systems into probiotic strains to enable targeted release of AhR ligands or SCFA precursors within the hypoxic colonic lumen. When combined with pH-responsive nanocarrier delivery platforms, this strategy enables site-specific and mucosa-targeted metabolite release, overcoming the functional uniformity and non-specificity of conventional probiotic therapies. Such approaches offer promising avenues for the development of personalized therapeutics against inflammatory diseases (Liu et al., 2023).

### 3.2 Remote immune regulation and systemic effects

Emerging evidence highlights that gut-derived metabolites convey immunomodulatory signals via the portal vein, lymphatic network, and neural circuits, thereby orchestrating immune regulation across distant organs such as the liver, lungs, and bone marrow (Kim, 2021). Upon entering the liver through portal circulation, SCFAs and SBAs engage nuclear receptors and G protein-coupled receptors (e.g., FXR and TGR5), initiating metabolic-immune signaling cascades in hepatocytes and hepatic immune cells. Notably, butyrate supports the polarization of liver macrophages toward the anti-inflammatory M2 phenotype, contributes to lipid regulation, and inhibits fibrotic progression in hepatic tissues (Yang et al., 2018; Mahalingam and Pandiyan, 2024). Lipophilic metabolites follow an alternative route, entering intestinal lymphatic vessels via chylomicron transport and reaching systemic circulation through the thoracic duct. This lymphatic pathway becomes particularly prominent under conditions such as compromised gut barrier function or high-fat dietary intake (Wastyk et al., 2021). In parallel, the gut-brain-immune axis has gained prominence as a remote regulatory system. SCFAs such as butyrate can activate vagal afferent neurons, thereby influencing the hypothalamic-pituitary-adrenal axis and sympathetic nervous output, ultimately shaping neuroimmune interactions (Silva et al., 2020). These effects extend beyond neurobehavioral regulation to encompass peripheral immune modulation. At the systemic level, these gut-derived metabolites significantly reshape immune cell populations and functions in peripheral organs. SCFAs facilitate the expansion of Tregs in the spleen and lungs by inhibiting HDACs and activating receptors such as GPR43 and GPR109A, effectively dampening Th1/Th17-mediated inflammatory responses (Liu et al., 2023). Additionally, in the bone marrow, SCFAs influence the differentiation trajectory of hematopoietic stem and progenitor cells, promoting the development of anti-inflammatory monocytes and myeloid lineages that enhance tissue repair following injury or infection (Wastyk et al., 2021).

Notably, this form of remote immune modulation exhibits distinct organ specificity and spatiotemporal dynamics. For example, during intestinal inflammation or metabolic stress, butyrate and its derivatives preferentially accumulate in the liver and lungs, where they regulate local immune homeostasis. In contrast, under steady-state conditions, their distribution is more diffuse, resulting in subtler immunomodulatory effects (Dalile et al., 2019). Moreover, host factors such as age, microbiota composition, and mucosal barrier integrity significantly impact metabolite bioavailability and immune targeting. Collectively, gut microbial metabolites coordinate a complex, multi-pathway, and multi-organ signaling network—referred to as the “metabolite-remote organ-immune response” axis—that expands our understanding of microbe-host interactions and reveals novel therapeutic targets for inflammatory, metabolic, and immune-related disorders.

### 3.3 Dose-dependent effects of metabolites

Gut microbial metabolites influence immune regulation in a dose- and time-dependent manner, a pattern often described as the “dose-time window” effect. While low concentrations typically promote immune balance and support tissue regeneration, elevated concentrations can lead to cellular stress or suppress immune function, underscoring their dualistic, “double-edged sword” nature. Therefore, achieving therapeutic benefits hinges on tightly controlled concentrations and the precise timing of metabolite release. Butyrate, a key SCFA, exemplifies this principle. At low doses (nanomolar to micromolar levels), it activates GPR109A receptors on intestinal epithelial cells, initiating autophagy and facilitating epithelial restoration. It also increases the localization of tight junction proteins such as claudin-3 at the cell membrane, thereby enhancing the integrity of the mucosal barrier (Faber et al., 2022). Within the T-cell milieu, low-dose butyrate suppresses HDAC3 activity, resulting in increased histone acetylation at the Foxp3 promoter and markedly promoting Treg differentiation—up to a 2.5-fold improvement (Hsu et al., 2024). However, when present at millimolar concentrations, butyrate triggers excessive mitochondrial reactive oxygen species generation in intestinal epithelial cells, activates BAX/BAK-dependent apoptosis, and elevates cell death rates above 70%, ultimately compromising barrier function and increasing intestinal permeability (Salvi and Cowles, 2021). This toxicity threshold is influenced by gut microenvironmental factors, including pH and microbial metabolic states. For instance, stem cells located at the crypt base in the proximal colon display relative resistance to elevated butyrate concentrations through the upregulation of silent information regulator two homolog 1, whereas differentiated epithelial cells at the villus tip exhibit greater sensitivity (Hsu et al., 2024). SBAs, such as DCA and LCA, support mucosal barrier integrity and immune homeostasis at physiological concentrations by activating the FXR and TGR5 receptors. However, pathological accumulation of SBAs induces oxidative stress, mitochondrial membrane potential loss, DNA strand breaks, proinflammatory responses, apoptosis, and oncogenic pathway activation (Hu et al., 2021). Animal models revealed a dose-dependent correlation between SBA accumulation induced by cholestasis or high-fat diets and hepatocellular carcinoma development. Clinical studies have reported that reduced DCA and LCA levels in the intestines of inflammatory bowel disease patients correlate with decreased Treg numbers and increased barrier permeability, underscoring their protective role in intestinal immune tolerance (Ju et al., 2023). The biological effects of SBAs are also influenced by organ- and spatial-specific distributions. SBA concentrations in the proximal colon typically range from 300 to 500 μM, where they primarily influence epithelial cells and local immune populations. In contrast, lower concentrations in the portal vein (5–10 μM) target hepatocytes and Kupffer cells. Individual SBA species exhibit a tissue-specific distribution; for example, LCA predominantly accumulates in the colon, whereas DCA is more likely to recirculate to the liver. This differential localization underpins their spatially distinct immunomodulatory and pathogenic roles (Devlin and Fischbach, 2015). AhR ligands also demonstrate time- and concentration-dependent regulatory functions. At physiological concentrations, IPA activates the AhR-STAT3 signaling axis, promoting IL-22 secretion and enhancing the expression of the antimicrobial peptide Reg3γ, thereby reinforcing the intestinal defense barrier (Bahman et al., 2024). Conversely, excessive IPA levels or sustained AhR activation increase IDO1 expression, leading to local tryptophan depletion and the apoptosis of T cells—particularly Th17 subsets, which can decrease by more than 80%. This shift fosters an immunosuppressive milieu and increases susceptibility to infection (Chen et al., 2023). Clinical evidence also suggests that AhR agonists such as laquinimod alleviate inflammatory bowel disease inflammation at low doses but can increase immune tolerance at high doses (Coskun et al., 2017). Polyamines at moderate levels maintain epithelial integrity and immune tolerance; however, within certain tumor microenvironments, they may promote tumor growth and immune evasion.

To address these dose-dependent challenges, recent advances have led to the development of sophisticated delivery and regulation strategies. pH gradient-responsive nanoparticles, such as chitosan-butyrate conjugates, enable slow release of low-dose butyrate in the ileum (pH 6.5–7.0) to promote repair and rapid high-dose release in the colon (pH 5.5–6.5) to induce local immune tolerance (Mahami et al., 2023). Moreover, sensor-engineered bacterial strains incorporating inflammation markers (e.g., IL-6 sensors) coupled with CRISPR-dCas9 regulatory circuits dynamically modulate IPA or SCFA precursor synthesis in response to the host inflammatory status, achieving a precise balance between immune activation and suppression (Wang et al., 2024). Engineered probiotics have also been applied for *in situ* synthesis or degradation of specific polyamines to reshape immune activity within the gut microenvironment.

## 4 Intestinal ecological balance and immune regulation

In a state of symbiosis, the gut microbiota exhibits high α diversity, a stable and balanced composition of commensal species, and an intact epithelial barrier maintained by tight junction proteins such as occludin, claudin-1, and ZO-1 (Sekirov et al., 2010). This stable ecosystem continuously produces a variety of bioactive metabolites, including SCFAs, SBAs, and tryptophan-derived indole compounds. Notably, these metabolites regulate both local and systemic immune responses through pathways such as the SCFA-PR41/43 signaling axis, FXR/TGR5 receptor activation, and the AhR-L-22 axis. They promote the differentiation of regulatory Tregs, increase IL-10 secretion, and suppress Th17-mediated inflammatory responses, thereby sustaining the delicate balance of mucosal and systemic immune tolerance (Visekruna and Luu, 2021).

**Figure 5:**
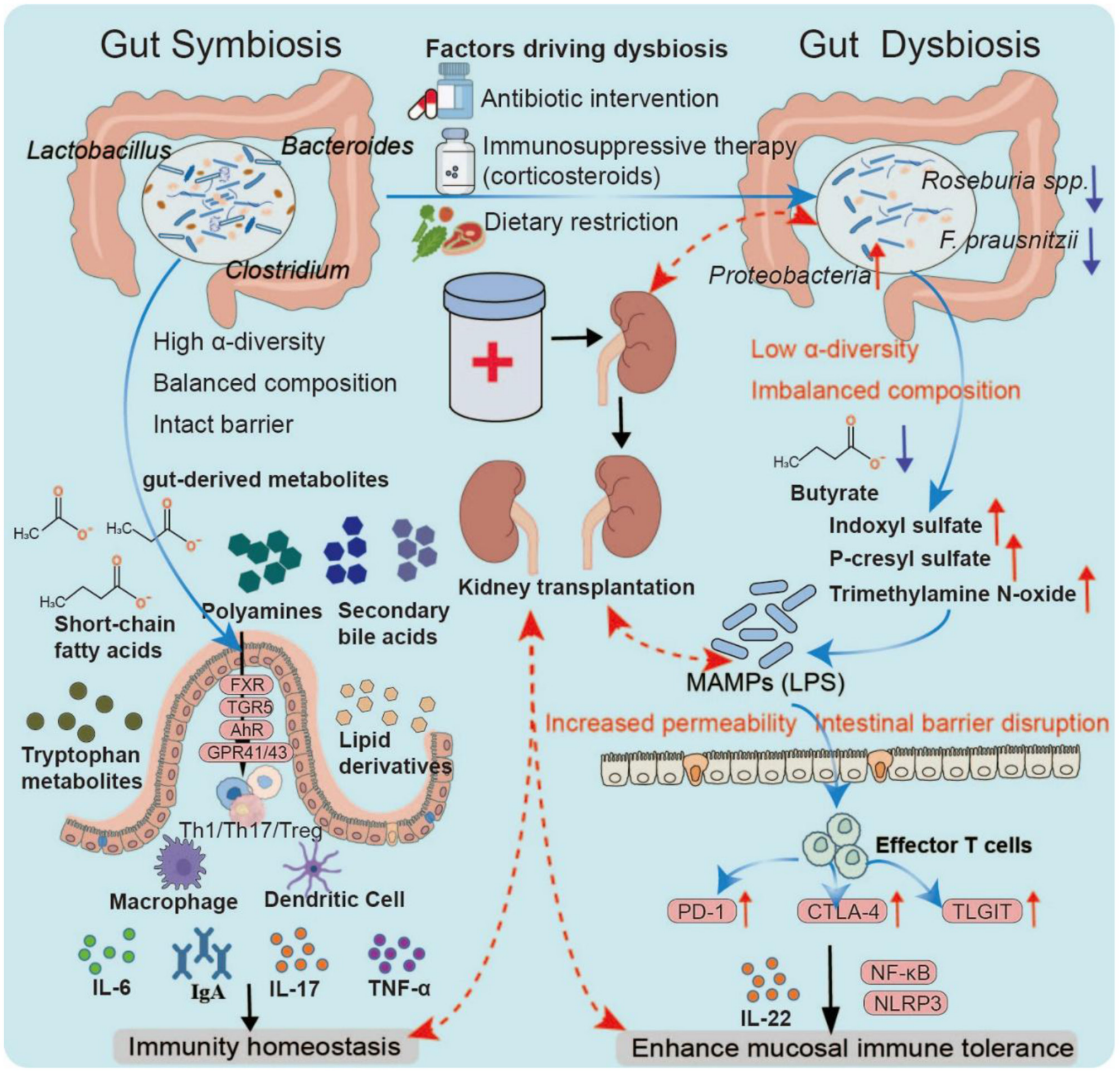
Gut dysbiosis and immune modulation. Under symbiosis, a diverse and balanced microbiota maintains barrier integrity and generates key metabolites that regulate immune pathways through receptors such as GPR41/43, FXR/TGR5, and AhR, thereby sustaining mucosal and systemic tolerance. Dysbiosis, often triggered by antibiotics, dietary restriction, or immunosuppressants, reduces diversity, depletes butyrate producers, and enriches Proteobacteria. This shift lowers protective metabolites while increasing uremic toxins, leading to barrier disruption and microbial product translocation. Antigen-presenting cell activation and immune checkpoint upregulation (PD-1, CTLA-4, and TIGIT) drive T-cell dysfunction and chronic inflammation, processes linked to impaired tolerance and adverse outcomes in transplantation.

Dysbiosis is a common yet underestimated factor in immune regulation (Kumar et al., 2022). It is characterized by a marked increase in Proteobacteria, a significant depletion of butyrate-producing bacteria (e.g., *F. prausnitzii, Roseburia* spp.), a metabolic shift from saccharolytic fermentation to proteolytic fermentation, and elevated serum concentrations of uremic toxins such as indoxyl sulfate, p-cresyl sulfate, and trimethylamine N-oxide. These toxins promote systemic inflammation and renal interstitial fibrosis (Evenepoel et al., 2009a; Rysz et al., 2021). Mechanistically, dysbiosis compromises epithelial barrier function, increases intestinal permeability, and facilitates the translocation of microbial-associated molecular patterns (MAMPs), such as LPS, into the bloodstream (Vaziri et al., 2013). MAMPs bind to pattern recognition receptors on antigen-presenting cells (APCs), activating downstream effector T cells and upregulating immune checkpoint molecules, including PD-1, CTLA-4, and TIGIT (Chen R. et al., 2025). Under physiological conditions, these checkpoints help limit excessive immune activation; however, in a chronically stimulated dysbiotic environment, they may lead to T-cell exhaustion or a breakdown of immune tolerance, thereby exacerbating alloreactive immune injury. In the clinical context of kidney transplantation, the incidence and impact of dysbiosis are further amplified. Multicenter studies have reported that about 65–80% of kidney transplant recipients exhibit significant alterations in gut microbiota composition within 3 months post-surgery (Artiles et al., 2023). The contributing factors include perioperative broad-spectrum antibiotics (which significantly reduce α-diversity), dietary restrictions, ischemia-eperfusion injury, and prolonged immunosuppressive therapy (e.g., tacrolimus, mycophenolate mofetil, and corticosteroids) (Swarte et al., 2020). Clinical data show that patients experiencing acute rejection have a Shannon diversity index more than 35% lower than that of stable recipients and a relative abundance of Enterobacteriaceae reaching up to 30% (compared with < 10% in controls) (Holle et al., 2025). Microbial metabolites are critical regulators of the microbiota-immune axis. Butyrate promotes Treg differentiation by inhibiting HDACs and activating GPR109A, whereas the tryptophan metabolite indole-3-propionic acid enhances mucosal immune tolerance through AhR-mediated IL-22 production (Furusawa et al., 2013a). In contrast, uremic toxins activate the NF-κB and NLRP3 inflammasome pathways, driving chronic inflammation and reducing graft survival (Evenepoel et al., 2009b) (Figure 5).

Based on these mechanistic insights, several microbiota-targeted therapeutic strategies are being explored in the transplant field. These include structured dietary interventions, engineered precision probiotics capable of producing butyrate or indole derivatives, bacteriophage-nanomaterial conjugates for selective pathogen eradication, exogenous supplementation with purified SCFAs or synthetic analogs, and personalized dietary modulation or fecal microbiota transplantation (Fowoyo, 2024; Zhou et al., 2020; Glorieux et al., 2023). Early trials have shown promise in restoring microbial diversity, lowering inflammatory markers (e.g., CRP and IL-6), and prolonging graft survival; however, large-scale, multicenter trials with long-term follow-up are still needed to confirm the efficacy and safety of these methods.

## 5 Regulatory strategies for the metabolism-immune axis

### 5.1 Nutritional intervention

Nutritional strategies aimed at modulating gut microbial metabolites have evolved from broad dietary adjustments to a more refined ecological adaptation paradigm centered on the “metabolism-immune axis.” Recent efforts have focused on the targeted enrichment of functionally relevant microbiota by supplementing specific substrates—such as prebiotics—to alter microbial metabolic activity and promote immune equilibrium. Indigestible carbohydrates such as inulin and resistant starch selectively promote the proliferation of butyrate-producing species, including *F. prausnitzii* and *Anaerostipes* spp., generating butyrate gradients ranging from 1 to 3 mM in the proximal colon to 0.5–1 mM in the distal colon. Clinical evidence indicates that administering 20 g of resistant starch daily for 4 weeks in Crohn's disease patients leads to a nearly two-fold increase in fecal butyrate levels, accompanied by a 35% increase in mucosal healing rates (Clarke et al., 2007). To address the rapid uptake and degradation of butyrate in the upper gastrointestinal tract, innovative postbiotic delivery technologies have emerged. Ethyl cellulose-butyrate conjugates with pH-sensitive properties allow for sustained, low-dose release in the ileum (pH 6.5–7.0) and prompt, high-dose release in the colon (pH 5.5–6.5), improving regional bioavailability. Furthermore, nanoliposomal formulations of butyrate, which achieve encapsulation efficiencies above 90%, have been shown in animal models to effectively penetrate the mucus barrier and be delivered to colonic crypt bases, resulting in an about 60% decrease in epithelial apoptosis in DSS-induced colitis (Ota and Sakuraba, 2022). In addition to butyrate, inulin-type fructans have been demonstrated to increase the abundance of *Anaerostipes* and *Agathobacter* species, thereby increasing butyryl-CoA:acetate CoA-transferase activity and further promoting SCFA production (Van-Wehle and Vital, 2024).

The emergence of synthetic biology has accelerated the development of “smart probiotics”—engineered microbial strains equipped with CRISPR-dCas9 systems that are responsive to inflammatory cytokines such as IL-6 (Lynch et al., 2022). These systems dynamically regulate the expression of IPA synthase, thereby maintaining AhR ligand concentrations within a physiological micromolar range and preventing overstimulation, which could lead to immunosuppressive outcomes (Luk-In et al., 2024). In parallel, advanced multiorgan-on-a-chip technologies incorporating intestinal, hepatic, and lymphoid tissue models enable synchronized simulation of metabolite transport across biological barriers and corresponding immune responses. These platforms support the precise quantification of metabolite effects under various concentration profiles and exposure timelines. For example, in cellular models derived from Crohn's disease patients, such systems establish an optimal local butyrate concentration range of 0.5–1.2 mM and corresponding exposure duration, providing a quantitative framework for tailoring nutritional interventions (Van-Wehle and Vital, 2024). Overall, the integration of targeted prebiotic supplementation, nanoscale delivery technologies, and synthetic biology-based responsive probiotics constitutes a multidimensional nutritional intervention strategy. This comprehensive framework holds significant promise for reprogramming the gut metabolism-immune interface in a personalized and physiologically attuned manner.

### 5.2 Microbial engineering

With recent advancements in synthetic biology, microbial engineering has emerged as a promising strategy for modulating the gut metabolism-immune axis. This approach centers on the development of engineered bacterial strains designed to synthesize and deliver immunomodulatory metabolites with precise spatial and temporal control within the intestinal milieu. When combined with phage-based strategies for the selective elimination of pathogenic microbes, this methodology signifies a paradigm shift from broad ecological modulation to targeted immunometabolic regulation. Recent efforts have focused on engineering *Escherichia coli* Nissle 1917 strains by incorporating synthetic operons capable of sensing intestinal inflammatory cues, such as thiosulfate and nitrate, and triggering the inducible biosynthesis of indole-derived metabolites. Under inflammatory conditions, these engineered strains increase the expression of indole-synthesizing enzymes, leading to the production of measurable levels of indole-3-acetic acid. Indole-3-acetic acid subsequently activates the host AhR-IL-22 signaling pathway, promoting the expression of antimicrobial peptides such as Reg3γ and facilitating epithelial tissue repair. Therapeutic efficacy has been demonstrated in models of chemically induced liver injury (Woo et al., 2025). In models of alcohol-related liver disease, *E. coli* Nissle 1917 strains engineered to constitutively express multiple AhR agonists effectively reduce hepatic inflammation and fibrosis. This therapeutic effect was significantly attenuated in IL-22-deficient mice, underscoring the pivotal role of the AhR-IL-22 axis in mediating host responses (Kouno et al., 2023). To ensure the dynamic, safe modulation of host-microbial interactions, CRISPR-dCas9 systems have been integrated into *E. coli* Nissle 1917 strains to establish a “sense-response” regulatory circuit. These constructs enable real-time adjustment of metabolic pathway activity in response to host cytokine fluctuations, whereas embedded biosafety modules limit long-term colonization and reduce ecological perturbation (Lynch et al., 2022). Additionally, by fusing YebF secretion signals with synthetic peptide tags, engineered strains have been developed to deliver effector molecules that target tumor-associated markers to colorectal cancer cells, expanding their utility in localized immune modulation and antitumor therapies (Ba et al., 2024). Moreover, phage therapy has re-emerged as a highly specific tool for pathogenic bacterial clearance with minimal impact on the commensal microbiota.

Targeted bacteriophages specific to Enterobacteriaceae strains with elevated LPS expression have proven effective in alleviating intestinal inflammation while maintaining the integrity of commensal microbial populations (Chen et al., 2023). By employing high-throughput microfluidic screening, researchers have curated a comprehensive “phage-host matching database” that enables rapid identification of host-specific phages from diverse gut bacterial isolates. An increase in mucosal penetration strategies has further improved phage viability and functional activity within the intestinal mucus environment (El Haddad et al., 2022). In early-stage clinical studies involving individuals with diabetes or inflammatory bowel disease, orally delivered, patient-tailored phage formulations significantly decreased Enterobacteriaceae colonization in mesenteric lymph nodes. This was accompanied by an about 35% reduction in systemic inflammatory markers, including interleukin-6 (IL-6), without appreciable disturbance to the beneficial gut microbiota (Chen et al., 2023).

To augment therapeutic precision and efficacy, phages have been conjugated with nanomaterials—such as polyethylene glycosylation and anti-LPS antibody fragments—enabling dual-targeting mechanisms and improved adherence to mucosal surfaces. Additionally, genetically engineered phages designed to express anti-inflammatory genes are capable of both lysing pathogenic bacteria and concurrently delivering immunoregulatory payloads. This dual-function design establishes a synergistic “lysis-anti-inflammatory” mechanism that has shown promise in reducing intestinal inflammation scores and attenuating host anti-phage immune responses in preclinical models (Kumar et al., 2025; Maurya et al., 2022; Vaaben et al., 2025; Tumas et al., 2023). Emerging delivery innovations, including pH-sensitive microcapsules and CRISPR-Cas9-mediated reprogramming of commensal metabolic pathways, offer novel avenues for microbiota-specific therapeutic interventions (Fiorucci et al., 2025). Overall, these synthetic biology-driven approaches harness the power of microbial engineering to achieve highly targeted metabolite delivery and pathogen clearance, enabling sophisticated spatiotemporal control over host metabolic and immune functions.

### 5.3 Drug development: spatiotemporal precision targeting of the metabolism-immunity axis

As the mechanistic understanding of the gut-immune-metabolic axis advances, pharmacological strategies targeting metabolic receptors are increasingly being refined for spatiotemporal precision. In recent years, integrated approaches combining small-molecule receptor modulators with nanocarrier-based delivery systems have emerged as promising modalities for modulating metabolic-immune interactions, particularly in the context of inflammatory and metabolic liver disorders. FXR agonists exemplify this paradigm. Obeticholic acid, an FXR agonist, is approved for the treatment of primary biliary cholangitis, where it mitigates inflammation associated with cholangitis primarily through inhibition of the NF-κB signaling cascade. Nevertheless, prolonged administration of obeticholic acid is often associated with systemic side effects, most notably pruritus, which can impair patient adherence and quality of life in up to 60% of cases (Morrison and Elgendy, 2024). To overcome these challenges, gut-restricted FXR agonists such as MET409 have been engineered. Owing to its high molecular weight and limited membrane permeability, MET409 demonstrates preferential gastrointestinal retention and reduced hepatic exposure. Phase III clinical trials have shown that MET409 improves fibrosis resolution in patients with non-alcoholic steatohepatitis while also resulting in a lower incidence of severe adverse events and an improved tolerability profile (Qu et al., 2023). Beyond receptor agonism, the targeted delivery of small-molecule ligands via nanotechnology-based platforms has shown considerable promise. A notable example is the encapsulation of the endogenous AhR agonist 6-formylindolo [3,2-b] carbazole within PEGylated liposomes conjugated to anti-CD4 antibodies, enabling selective delivery to CD4^+^ T cells within the intestinal lamina propria. Similarly, the TLR4 antagonist TAK-242 was formulated into a liposomal preparation for hepatic targeting. A similar strategy has also been used for IL-22 delivery, with IL-22-encoding plasmids loaded on pH-responsive nanoparticles and orally delivered for local desensitization release in the colon, resulting in efficient repair of the intestinal epithelium and significant improvement in pathological scores in a mouse model of colitis (Qu et al., 2023). When administered via the portal vein, TAK-242 preferentially accumulates in the liver sinusoidal region, inhibits the TLR4/MyD88 signaling axis, and reduces IL-1β and TNF-α secretion (Li et al., 2025). Optimization of the nanoparticle size and surface charge (slightly negative) increased hepatic specificity and retention while minimizing off-target toxicity. Notably, multi-responsive nanocarriers are facilitating the development of “regionalized controlled release” strategies. For example, dual-gradient-responsive nanocrystals have been designed to release low doses of FXR agonists within the intestinal lumen in response to pH variations, followed by disintegration in the hypoxic hepatic microenvironment to deliver relatively high concentrations of anti-inflammatory agents. This approach enables finely tuned spatial and temporal drug distribution along the gut-liver axis (Elkordy et al., 2024). When coupled with synthetic biology-engineered “sense-and-respond” probiotic strains, such systems offer dynamic modulation of drug dosage and therapeutic windows in accordance with local microenvironmental signals.

In summary, the convergence of receptor-targeted small molecules and sophisticated nanodelivery platforms is shaping a novel paradigm for precise regulation of the gut-liver-immune axis. This integrated strategy holds substantial potential to enhance therapeutic outcomes while mitigating systemic side effects, marking a pivotal advancement in the design of next-generation immunometabolic therapies.

## 6 Conclusion

Gut microbial metabolites play a key role at the host immune interface, affecting immune cell differentiation, cytokine production, and barrier integrity through multiple signaling pathways. Key metabolite classes, including SCFAs, SBAs, Trp, polyamines, and lipid-derived mediators, exert both proinflammatory and anti-inflammatory effects to shape immune homeostasis in health and disease. Advances in microbial engineering, dietary modulation, and metabolite-based therapeutics offer promising avenues for targeting immune modulation.

However, significant challenges remain, such as individual differences in microbiome composition, complex interactions between microbial metabolism and host genetics, and the influence of environmental factors complicating translation to clinical practice. The bidirectional relationship between gut dysbiosis and immune rejection in transplantation medicine, particularly kidney transplantation, underscores the need for integrated strategies that combine microbiome analysis with immune surveillance. Future research should focus on mechanistic studies linking specific microbial pathways to definitive immune outcomes, the development of precision microbiome therapies, and the incorporation of metabolomics and metagenomics tools into personalized medicine. By connecting the basic microbiome with translational immunology, gut microbial metabolites can be further exploited to improve immune-related disease outcomes and long-term transplant success.

## 7 Challenges and future perspectives

The gut microbiota-immune axis represents a promising frontier for therapeutic innovation, yet its clinical translation, particularly in kidney transplantation, remains constrained by several unresolved challenges. In healthy individuals, the symbiotic microbiota sustains immune homeostasis via metabolite-mediated pathways, such as SCFA-GPR41/43 signaling, bile acid-FXR/TGR5 activation, and tryptophan-AhR modulation. In contrast, immunosuppressive regimens in kidney transplant recipients (e.g., tacrolimus, mycophenolate mofetil, and corticosteroids) frequently induce dysbiosis, characterized by reduced SCFA production, enrichment of *Proteobacteria*, and depletion of key commensals such as *F. prausnitzii*.

Key obstacles include the mechanistic complexity of the gut-immune axis, wherein overlapping pathways may exert distinct effects under immunosuppression; substantial interindividual variability in microbial composition driven by genetic, dietary, environmental, and pharmacological factors; unclear interactions between dysbiosis and immune checkpoint molecules (programmed death-1, cytotoxic T lymphocyte-associated protein-4, T-cell immunoglobulin and the ITIM domain), which may influence graft tolerance or rejection; and the scarcity of large-scale, longitudinal human studies linking specific metabolite profiles to transplant outcomes.

Future research should prioritize precision microbial engineering to generate targeted immunomodulatory metabolites (e.g., butyrate, indole-3-propionic acid); the development of phage-nanomaterial conjugates to selectively eliminate pathogenic strains; metabolite-based therapies using purified compounds or synthetic analogs to restore immune balance without altering the global microbiota composition; the integration of multiomics approaches for personalized microbiome-immune risk profiling; and the development of transplant-specific interventions such as tailored diets, probiotic consortia, or fecal microbiota transplantation. A deeper understanding of the dysbiosis-immune rejection axis will be critical for advancing microbiota modulation from an experimental concept to a standardized component of post-transplant care.
